# Utility of quantitative contrast-enhanced ultrasound for the prediction of extracapsular extension in papillary thyroid carcinoma

**DOI:** 10.1038/s41598-017-01650-2

**Published:** 2017-05-03

**Authors:** Yi Liu, Hua Liu, Chang-Lin Qian, Mei-Sui Lin, Feng-Hua Li

**Affiliations:** 1 Departments of Ultrasound, South Campus, Renji Hospital, School of Medicine, Shanghai Jiao Tong University, 2000 Jiangyue Road, Shanghai, 201112 China; 2 Departments of General Surgery, South Campus, Renji Hospital, School of Medicine, Shanghai Jiao Tong University, 2000 Jiangyue Road, Shanghai, 201112 China; 3 Departments of Pathology, South Campus, Renji Hospital, School of Medicine, Shanghai Jiao Tong University, 2000 Jiangyue Road, Shanghai, 201112 China

## Abstract

The aim of this study was to find an accurate method for the detection of extracapsular extension (ECE) in papillary thyroid carcinoma (PTC). A total of 102 patients with 109 PTC nodules were retrospectively enrolled. Contrast-enhanced ultrasound (CEUS) characteristics were evaluated. The diagnostic efficacy of quantitative CEUS and tumor size was analyzed. The qualitative CEUS features did not differ significantly between the ECE and non-ECE groups (*P* > 0.05). All of the quantitative CEUS parameters with the exception of peak intensity and tumor size were found to differ significantly between the ECE and non-ECE groups (*P* < 0.05). Multivariate stepwise logistic regression analysis demonstrated that time from peak to one half (TPH), tumor size and wash-in slope (WIS) were the significantly different parameters between the ECE and non-ECE groups (*P* = 0.000, *P* = 0.005 and *P* = 0.030, respectively).The sensitivity and specificity in the diagnosis of ECE were: TPH, 75.4% (43/57) and 78.9% (41/52), respectively; WIS, 87.7% (50/57) and 42.3% (22/52), respectively; and tumor size, 71.9% (41/57) and 65.4% (34/52), respectively. Quantitative CEUS analysis and tumor size are essential for the prediction of ECE in PTC; in particular TPH has good diagnostic value in detecting ECE. Our study provides important insights into the prediction of ECE in PTC.

## Introduction

Recently, the incidence of thyroid cancer has increased sharply. Papillary thyroid carcinoma (PTC) is the most common pathologic type of thyroid cancer. A number of PTC patients have a favorable prognosis. However, some PTC patients have local recurrences in the neck. Thyroid extracapsular extension (ECE) is strongly related to the local recurrence of PTC in the neck^1–3^. ECE has an important role in determining the tumor stage and extent of thyroid surgery in patients with PTC. PTC patients with ECE always have a worse prognosis than those without ECE^4, 5^.

**Figure 1:**
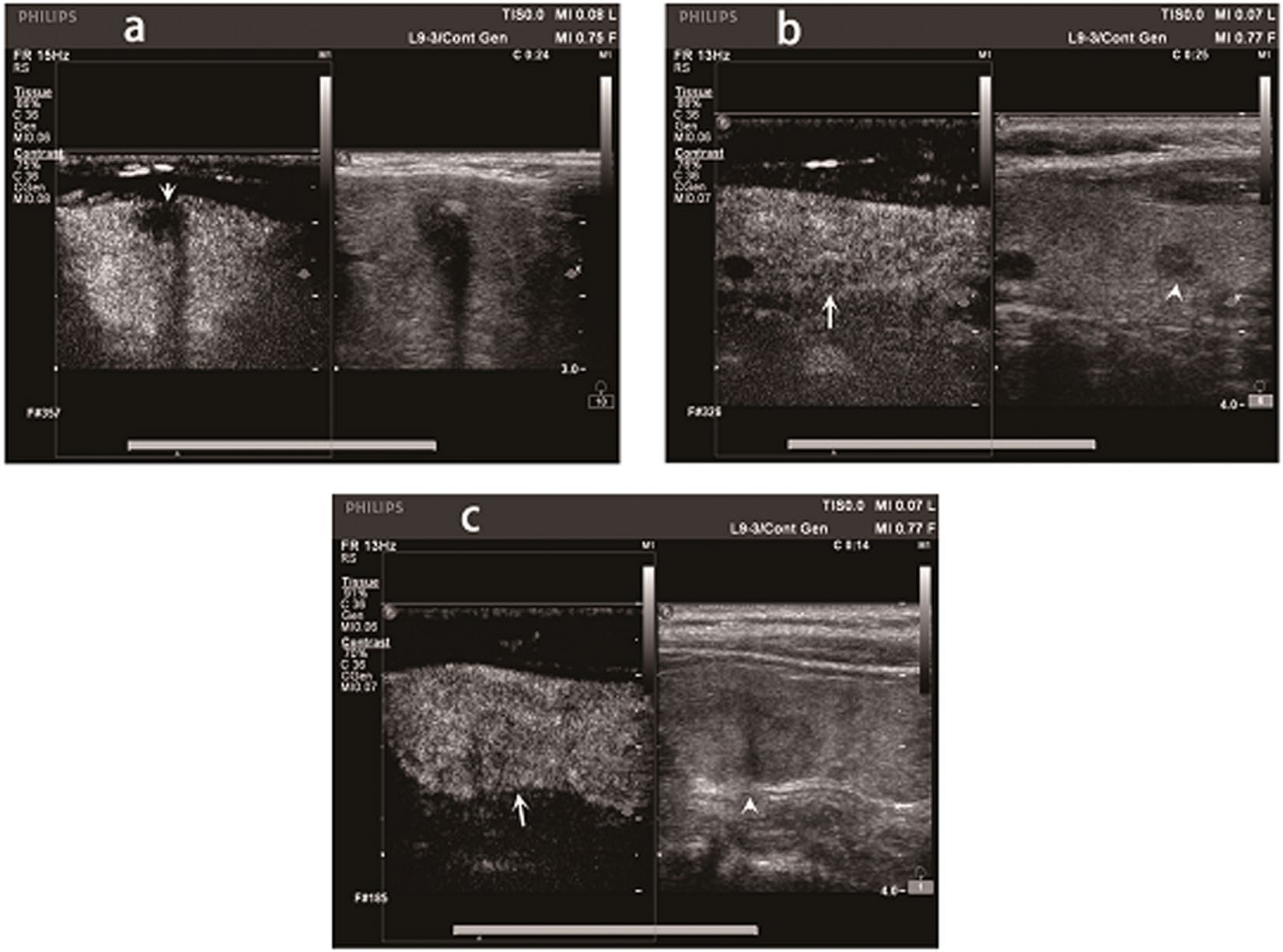
The Contrast-enhanced ultrasound (CEUS) images of different papillary thyroid carcinoma (PTC) patients with extracapsular extension (ECE). (**a**) CEUS image of a 48-year-old male PTC patient with ECE. The CEUS image clearly showed that the anterior thyroid capsular was invaded by PTC (arrow). (**b**) CEUS image of a 58-year-old female PTC patient with ECE. The nodule showed by arrow head was the PTC nodule. ECE was not clearly confirmed by CEUS (arrow). (**c**) CEUS image of a 50-year-old female PTC patient with ECE. The nodule showed by arrow head was the PTC nodule. ECE was not clearly detected by CEUS (arrow).

Ultrasonography (US), multi-slice computed tomography and magnetic resonance imaging are the imaging techniques used for the detection of thyroid cancers. However, US has limited accuracy and sensitivity in the detection of ECE^6–9^. Doh Young Lee *et al*.^9^ stated that the positive predictive value (PPV) of computed tomography (CT) concerning the detection of ECE in PTC patients was higher than that of US. This suggested that CT could predict ECE more accurately than US. US elastography (USE) has been proposed for the differential diagnosis of benign and malignant lesions according to the tissue stiffness, such as thyroid nodule differential diagnosis^10^. The study of Magri F^10^ reported that the elastographic strain index had a high sensitivity, specificity, and negative predictive value (NPV) for identifying thyroid malignancy. Contrast-enhanced ultrasonography (CEUS) is a promising tool in the study of microvascular flow in different organs. CEUS is also very helpful in differentiating benign and malignant tumors^11–14^. Recently, CEUS has been reported to improve the sensitivity and specificity of US in the diagnosis of ECE in PTC patients. Xi Wei *et al*.^15^ concluded that the sensitivity 91.1% (62/68) and specificity 86.5% (45/52) of CEUS were higher than those of US in the diagnosis of ECE (49%, 120/242 and 55%, 70/127 respectively). CEUS could contribute to the diagnosis of ECE in PTC and have utility in the surgical management of PTC patients. In this study, PTC patients with ECE all exhibited hypoenhancement and well-defined margins regarding CEUS features. However, PTC patients with ECE could show atypical CEUS features, for example, isoenhancement, hyperenhancement and ill-defined margins. The diagnosis of ECE exclusively using conventional CEUS remains unclear and has limitations (Fig. 1). Hence, a comprehensive method of estimation rather than simple conventional CEUS could be beneficial for the diagnosis of ECE. We therefore hypothesize that there may be some differences in the quantitative and qualitative features of CEUS between ECE and non-ECE groups. At present, few reports have dealt with these features in the prediction of ECE in PTC patients. Thus, our aims were to develop a more accurate method to predict ECE preoperatively in PTC patients and to decide the extent of surgery.

## Results

### Patient demographic characteristics

Patient characteristics are detailed in Table 1. Fifty-seven nodules (57/109 = 52.3%) were diagnosed as ECE and 52 nodules (52/109 = 47.7%) as non-ECE by means of histopathology. Patients with ECE had a significantly larger tumor size than patients without ECE (12.7 ± 7.8 mm vs 8.2 ± 3.5 mm; *P* = 0.000), while patient age and gender did not differ significantly between ECE and non-ECE groups (*P* = 0.469 and *P* = 0.591, respectively).

**Table 1:** Patients characteristics of ECE and non-ECE group.

Characteristics	ECE	non-ECE	*P* value
Gender			
Male	10	8	0.591
Female	43	43	
Nodule No.	57	52	
Age (yrs)	48.7 ± 12.8	46.9 ± 12.2	0.469
Tumor size (mm)	12.7 ± 7.8	8.2 ± 3.5	0.000

ECE: extracapsular extension.

### Qualitative CEUS features of ECE and non-ECE patients

Our study found that the degree of contrast agent distribution, border type and degree of enhancement did not differ significantly between the ECE and non-ECE groups (*P* > 0.05, for all parameters) (Table 2).

**Table 2:** Comparison of CEUS qualitative and quantitative parameters between ECE and non-ECE group.

Parameters	Characteristics	ECE		*P* value
		Yes (n = 57)	No (n = 52)	
The degree of contrast agent distribution	*Diffuse heterogeneous enhancement*	28	22	
	*Diffuse homogeneous enhancement*	9	10	0.762
	*Dotted enhancement*	20	20	
Border type	*Well-defined border*	23	23	0.682
	*Ill-defined border*	34	29	
Enhancement degree	*Hypoenhancement*	47	42	
	*Isoenhancement*	9	9	0.975
	*Hyperenhancement*	1	1	
RT(sec)		6.86 ± 5.33	9.92 ± 8.51	0.025
TTP(sec)		23.07 ± 8.42	27.67 ± 11.07	0.016
PI(dB)		7.67 ± 2.14	7.89 ± 2.16	0.593
WIS(dB/sec)		1.27 ± 0.59	0.91 ± 0.61	0.003
TPH(sec)		51.72 ± 15.33	76.85 ± 24.55	0.000
AUC(dBsec)		541.35 ± 200.87	675.16 ± 196.58	0.001

CEUS: contrast-enhanced ultrasound. ECE: extracapsular extension. RT: rise time. TTP: time to peak. PI: peak intensity. WIS: wash in slope. TPH: time from peak to one half. AUC: area under the curve.

### Quantitative CEUS parameters for ECE and non-ECE patients

**Figure 2:**
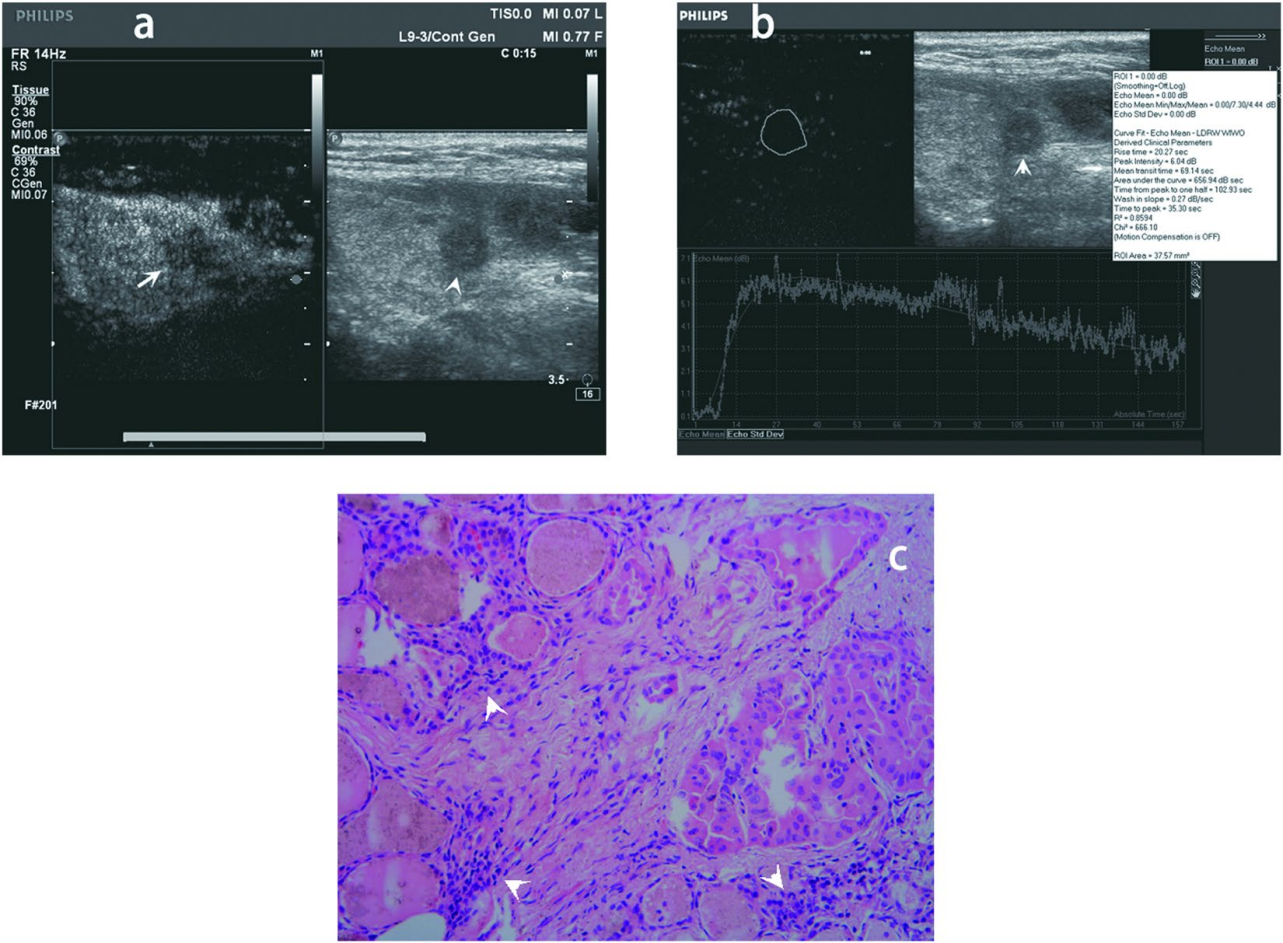
Qualitative and quantitative contrast-enhanced ultrasound (CEUS) image and histopathologic image of a 57-year-old female papillary thyroid carcinoma (PTC) patient without extracapsular extension (ECE). (**a**) Qualitative CEUS image of the patient. The PTC nodule in gray scale ultrasound image was showed by arrow head. The PTC nodule in CEUS image was showed by arrow. (**b**) Quantitative CEUS image of the patient. Time-intensity curve showed rise time 20.27 sec, peak intensity 6.04 dB, area under the curve 656.94dBsec, time from peak to one half 102.93 sec, wash in slope 0.27dBsec, time to peak 35.30 sec. (**c**) Histopathologic image revealed PTC of the patient without ECE. The cancer cells were showed by arrow head (original magnification, x100).

**Figure 3:**
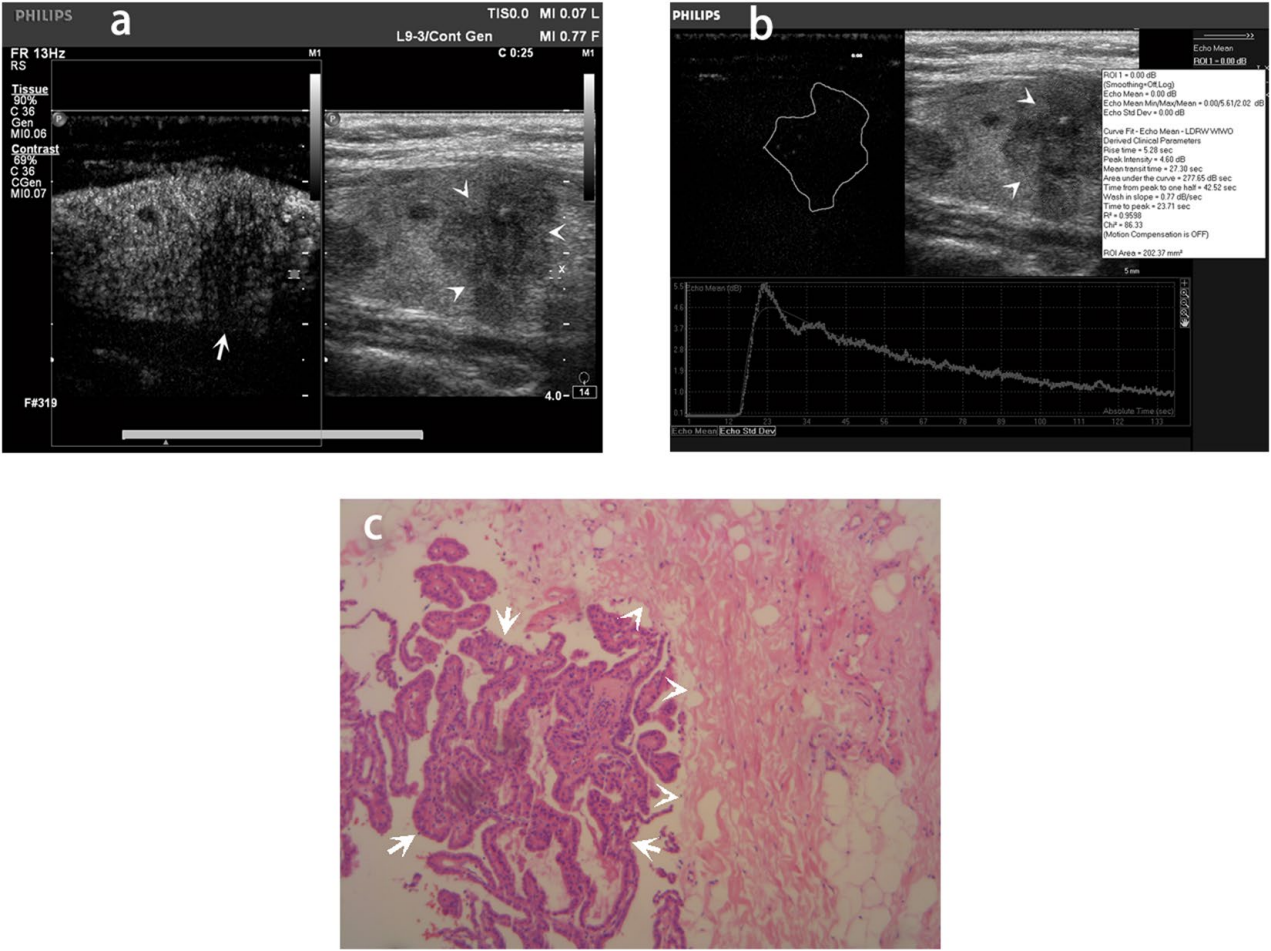
Qualitative and quantitative contrast-enhanced ultrasound (CEUS) image and histopathologic image of a 55-year-old female papillary thyroid carcinoma (PTC) patient with extracapsular extension (ECE). (**a**) Qualitative CEUS image of the patient. The PTC nodule in gray scale ultrasound image was showed by arrow head. The PTC nodule in CEUS image was showed by arrow. (**b)** Quantitative CEUS image of the patient. Time-intensity curve showed rise time 5.28 sec, peak intensity 4.60 dB, area under the curve 277.65dBsec, time from peak to one half 42.52 sec, wash in slope 0.77dBsec, time to peak 23.71 sec. (**c**) Histopathologic image revealed PTC of the patient with ECE. The cancer cells were showed by arrow. The thyroid capsule was showed by arrow head. The cancer cells breaked and grew outside the thyroid capsule (original magnification, x100).

As shown in Table 2, Figs 2 and 3, all of the quantitative parameters except for PI were found to be significantly different between the ECE and non-ECE groups. The WIS was significantly higher in the ECE group than in the non-ECE group (*P* = 0.003), whereas the RT, TTP and TPH were significantly shorter in the ECE group than in the non-ECE group (*P* = 0.025, *P* = 0.016 and *P* = 0.000, respectively); the AUC of the TIC was significantly smaller in the ECE group than in the non-ECE group (*P* = 0.001). The RT, TTP and WIS were all variables used to calculate wash-in. The TPH was the variable used to calculate wash-out. The shorter RT and TTP indicate rapid filling-in. The higher WIS indicates rapid wash-in and the shorter TPH indicates rapid wash-out.

### Multivariate logistic regression analysis

Multivariate logistic regression analysis revealed that TPH (OR = 0.905; 95% confidence interval [CI], 0.863–0.949; *P* = 0.000), tumor size (OR = 1.176; 95% CI, 1.049–1.318; *P* = 0.005), and WIS (OR = 0.270; 95%CI, 0.083–0.882; *P* = 0.030) differed significantly between the ECE and non-ECE groups. Based on the above data, TPH was found to be the most significantly different parameter (Table 3).

**Table 3:** Multivariate logistic analysis of the CEUS and US parameters.

Parameters	OR	95%CI	*P* value
Tumor size	1.176	1.049–1.318	0.005
TPH	0.905	0.863–0.949	0.000
WIS	0.270	0.083–0.882	0.030

CEUS: contrast-enhanced ultrasound. US: ultrasound. WIS: wash in slope. TPH: time from peak to one half. OR: odds ratio. CI: confidence interval.

### Diagnostic performances of quantitative CEUS parameters and tumor size

**Figure 4:**
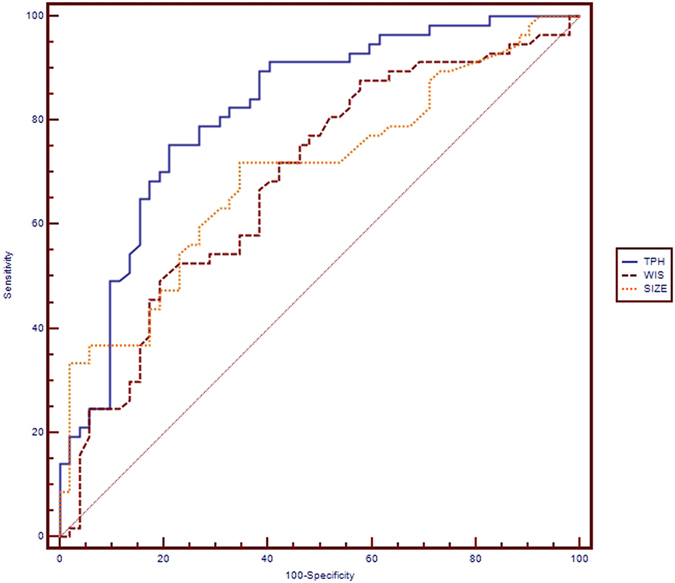
Receiver operating characteristic (ROC) curves of contrast-enhanced ultrasound quantitative parameters and tumor size for diagnosis of extracapsular extension (ECE) in papillary thyroid carcinoma. Time from peak to one half (TPH) (area under the curve 0.817, cut-off score 57.97 sec), wash in slope (WIS) (area under the curve o.685, cut-off score 0.64 dB/sec) and tumor size (area under the curve 0.699, cut-off score 7.6 mm). It suggests diagnostic significance of TPH, WIS and tumor size with area under the curve >0.5. The cut off score is choosed as that closest to the point of ROC curves with both highest sensitivity and specificity. A nodule with a measurement above the cut-off score of WIS or tumor size was regarded as ECE, and below the cut-off score of TPH was regarded as ECE.

The sensitivity, specificity, accuracy, PPV, NPV and AUC of CEUS quantitative parameters and tumor size were calculated regarding the diagnosis of ECE (Table 4). The pathologic diagnosis was considered as the gold standard. A receiver-operating-characteristic (ROC) curve analysis was performed to determine the cut-off scores for CEUS and US parameters in further characterizing the ECE of the PTCs. The cut-off score was the one closest to the point with both highest sensitivity and specificity (MedCalc® software). The AUC (0.817) for TPH was larger than that for WIS and tumor size (Fig. 4). The accuracy of TPH (77.1%, 84/109) was higher than that of WIS and tumor size. A nodule with a measurement above the cut-off score of WIS (0.64 dB/sec) or tumor size (7.6 mm) was regarded as ECE, and a nodule with a measurement below the cut-off score of TPH (57.97 sec) was regarded as ECE. The sensitivity and specificity of TPH in the diagnosis of ECE were 75.4% (43/57) and 78.9% (41/52) respectively, 87.7% (50/57) and 42.3% (22/52), respectively for WIS, and 71.9% (41/57) and 65.4% (34/52), respectively for tumor size.

**Table 4:** Diagnostic performances of different parameters to predict ECE in PTC patients.

Parameters	Sensitivity(%)	Specificity(%)	Accuracy(%)	PPV(%)	NPV(%)	Cut-off score	AUC
TPH	75.4 (43/57)	78.9 (41/52)	77.1 (84/109)	79.6 (43/54)	74.6 (41/55)	57.97 sec	0.817
WIS	87.7 (50/57)	42.3 (22/52)	66.1 (72/109)	62.5 (50/80)	75.9 (22/29)	0.64 dB/sec	0.685
Tumor Size	71.9 (41/57)	65.4 (34/52)	68.9 (75/109)	69.5 (41/59)	68.0 (34/50)	7.6 mm	0.699

ECE: extracapsular extension. PTC: papillary thyroid carcinoma. TPH: time from peak to one half. WIS: wash in slope. PPV: positive predictive value. NPV: negative predictive value. AUC: area under the curve.

## Discussion

Detection of the presence of ECE would be of great value in deciding the extent of surgery involving a change from lobectomy to total or near-total thyroidectomy. Total or near-total thyroidectomy could have higher complication rates than lobectomy and does not improve the treatment outcome and patient survival rate^16–18^. Therefore, the accurate prediction of ECE preoperatively would be very important in avoiding unnecessary surgical resection in PTC patients. The present study suggested that the quantitative CEUS parameters and tumor size were distinctly different in the ECE and non-ECE PTC patients, especially regarding the three parameters TPH, WIS and tumor size. Furthermore, the analysis of the diagnostic performance of these three parameters could be used to characterize ECE and non-ECE patients.

Measurement values from the TIC can be helpful if ECE is hard to diagnose precisely based on visual characteristics using CEUS. In the present study, PTC patients in the ECE group demonstrated a shorter TTP, RT and TPH, and a higher WIS than those in the non-ECE group. The shorter RT and TTP indicate rapid filling-in. The higher WIS indicates rapid wash-in and the shorter TPH indicates rapid wash-out. It is known that RT and TTP are mainly affected by the blood flow rate. The faster the blood flow rate, the shorter the TTP and RT, and WIS has also been shown to correlate with neo-arterisation. In addition, because of large neocapillaries, a large amount of contrast agent would centralize in the tumor, thus resulting in a steeper WIS^19^. Meanwhile, PTC tumors in patients with ECE exhibit faster wash-out than in the non-ECE group. Some studies have revealed the possible mechanisms in other organs^20, 21^. Yang *et al*.^20^ stated that in early primary hepatocellular carcinoma (HCC) the portal vein vascular supply was larger, so the wash-out time was lengthened. While in metastatic HCC, the tumor arterial vascular supply was larger, and thus the wash-out time was generally shorter. Hence, we postulated that in the ECE group in our study, there was higher blood perfusion and larger tumor arteries than in the non-ECE group; this led to a shorter RT and TTP, a steeper WIS and a quicker wash-out.

Previous studies have shown that quantitative CEUS parameters were strongly associated with the aggressiveness of malignant tumors. Botond *et al*.^22^ demonstrated that in invasive breast cancers, there was a statistically significant correlation between TTP and tumor grade (*P* = 0.023), progesterone receptor (PR) status (*P* = 0.042) and axillary node status (*P* = 0.025). The washout ratio measured at 21 s was strongly related to estrogen receptor status (*P* = 0.042) and PR status (*P* = 0.026). High pathologic grade (grade III) invasive breast cancer always exhibited earlier wash-in and accelerated wash-out, whereas low pathologic grade (grade I) invasive breast cancer always exhibited slower wash-in and delayed wash-out. When a tumor is more aggressive, its angiogenic activity will be increased. Faster accumulation of microvessels may result in the development of more arterio-venous shunts. Therefore, tumors with higher blood perfusion are more likely to have a higher histological grade. In some aspects, our findings were consistent with this concept. ECE could reflect the aggressiveness of PTC tumors. In the present study, it is possible that altered higher perfusion of PTC tumors in the ECE group resulted in faster contrast agent uptake (wash-in) and quicker contrast agent elimination (wash-out) than those in the non-ECE group.

However, different from previous studies, in the current study, we used multivariate stepwise logistic regression analysis to further characterize ECE and non-ECE patients, and ROC curves were used to select cut-off scores for the parameters. Multivariate regression statistical analysis revealed that TPH, WIS and tumor size were the strongest predictors of ECE. The ROC curves indicated the high accuracy and AUC of TPH, and the low specificity of WIS; TPH was the most valuable measurement approach with both high sensitivity and specificity regarding the diagnosis of ECE. The reasons for this are not fully understood. Murphy-Lavallee *et al*.^23^ concluded that quick wash-out in metastatic tumors may be associated with an increasing number of arterial vascular and draining veins, and the features of the microvasculature. Therefore, we postulated that perhaps in the ECE group of PTC patients, a change in the microvasculature would come into being. The number of arterial vascular and draining veins would increase massively, leading to the shorter TPH in the ECE group. Importantly, our present findings actually revealed that quantitative CEUS analysis could achieve a much more accurate diagnostic result in ECE as compared with conventional CEUS.

Our study had several limitations. First, the association between USE and ECE was not evaluated in the study. Investigation of the relationship between USE and ECE may have important value. Therefore, the further studies on the correlation between USE and ECE are needed in the future. Second, we exclusively evaluated ECE using quantitative CEUS analysis. The aggressive behavior of PTC includes lymph node metastases, recurrence and distant metastases. Hence, a larger series study including investigation of the relationship of other types of aggressive behavior of PTC using quantitative CEUS analysis is needed. Third, long-term follow-up was limited in our study. We should perform a prospective study including long-term follow-up. Fourth, selection bias may have occurred. All patients were diagnosed as having PTC and were enrolled in this study preoperatively mainly on the basis of suspicious US images. We should avoid this bias in a future study. Finally, the relatively small sample size could affect the accuracy of the present study. Consequently, a multi-center study is required in the future.

In conclusion, quantitative CEUS parameters and tumor size may have the potential to predict the presence of ECE in PTC; in particular, TPH has a high sensitivity and specificity in the diagnosis of ECE. TPH could be a useful marker in predicting and characterizing ECE. This finding could help surgeons to decrease the number of total thyroidectomies and to avoid high rates of surgical complications.

## Methods

### Study population

This retrospective study was approved by the local institution’s ethics committee. All patients were provided written informed consent. The methods in our study were carried out according to the Declaration of Helsinki^24^. From July 2014 to October 2016, a series of 118 patients underwent thyroid surgery on the basis of suspicious US findings and/or US-guided fine-needle aspiration biopsy (FNAB) outcome. They were all examined using conventional US and CEUS before surgery. The inclusion criteria were: presence of a nonfunctional (“cold”) thyroid nodule; suspicious malignant thyroid nodules at conventional US and scheduled for surgery; if the patients had more than 2 malignant nodules, 2 nodules that were most likely malignant were selected; a lesion diameter >0.5 cm (for lesions <0.5 cm, it was difficult to maintain the same imaging plane in CEUS because of breathing and arterial pulsations); and age >18 years. The contraindications were: a multinodular thyroid goiter or functional (“hot”) thyroid nodules; severe cardiopulmonary insufficiency; previous side effects during CEUS; pregnancy; and breastfeeding. US-guided FNAB was performed in 67 of 118 patients with 71 nodules. FNAB results were: 59 nodules were PTC, 7 nodules were suspected PTC, 3 nodules were indeterminate cytology and 2 nodules were not malignant. Another 51 patients didn’t undergo FNAB preoperatively for the reasons of unwillingness to perform FNAB or having the contraindications of FNAB. Of the 104 pathologically proved PTC patients, two patients were excluded because of the poor image quality. A total of 102 patients (18 men and 84 women) aged 21–80 years (mean, 48 years) with 109 PTC nodules were consecutively enrolled in this study. Eighteen males aged 28–64 years (mean, 41 years). Eighty-four females aged 21–80 years (mean, 49 years). Among them, three famales had two PTC nodules for each patient with one nodule in ECE group and the other in non-ECE group. Three famales and one male had two PTC nodules in ECE group for each patient. So a total of 95 patients had a solitary PTC nodule. ECE was confirmed by pathology; the enrolled patients were separated into an ECE group and a non-ECE group.

### Conventional US and CEUS examination

Conventional US was performed using a L12–5 transducer and CEUS was performed using a L9-3 transducer (Philips iU22 system: Philips, Bothell, WA, USA). The thyroid nodule and the lymph nodes in the neck were evaluated using gray-scale US and color Doppler US. Then the transducer was switched to CEUS mode for examination. The lesion’s largest plane in long axis was selected for CEUS. The focus was placed deeper than the nodule plane. The contrast medium was SonoVue (Sulphurhexafluoride: Bracco, Milan, Italy). A contrast agent was injected as an intravenous bolus in a 2.4 mL suspension through the antecubital vein followed by a 5 mL normal saline solution flush. The CEUS parameters were used with a frame rate of 15 Hz and a mechanical index of 0.07. The real-time CEUS images were observed continuously for 180 s and stored on the hard disk of the scanner in DICOM format. All images were analyzed independently by two experienced radiologists, especially in thyroid CEUS (Y.L. and F.H.L., with 6 years and 10 years experience in thyroid CEUS). They were blinded to the patient clinical data and histological findings. When there were different opinions in the image analysis, they will review the images togetherly and consult more experienced radiologist (with 15 years more experience in thyroid CEUS), finally they will come to an agreement.

### Analysis of qualitative CEUS pattern features

The qualitative CEUS patterns were as follows. (1) The degree of contrast agent distribution was separated into diffuse heterogeneous enhancement, diffuse homogeneous enhancement and dotted enhancement. Diffuse heterogeneous enhancement, diffuse homogeneous enhancement and dotted enhancement were identified as various diffuse enhancement echo levels, a diffuse and single enhancement echo level and faint or separate spots of contrast agent of the lesion after the injection of Sonovue respectively. (2) The border type was classified into a well-defined and an ill-defined border. (3) The degree of enhancement was divided into hypoenhancement, isoenhancement and hyperenhancement. Hypoenhancement, isoenhancement and hyperenhancement occurred when the lesion echogenicity was lower, equal and higher with respect to the normal thyroid parenchyma respectively.

### Analysis of quantitative CEUS parameters

Q-LAB software (Philips Medical Systems, Bothell, WA, USA) was performed to analyze the quantitative CEUS parameters. The region of interest (ROI) was drawn in the interior of the whole nodule. The signal intensity was expressed in decibels (dB). Quantitative CEUS parameters for the time-intensity curve (TIC) were obtained as follows: the peak intensity (PI) calculated as the TIC curve’s maximum intensity minus the curve’s baseline intensity; the rising time (RT) was measured as the time needed to increase from 5% of PI to 95% of PI; the time to peak (TTP) was the time needed to reach PI; the wash-in slope (WIS) was measured by subtracting 5% of the PI from 95% of the PI, then dividing it by the RT; the time from peak to one half (TPH) measured as the time needed from PI to when it drops to half of PI; and the area under the TIC (AUC).

### Statistical analysis

Statistical analyses were calculated using SPSS version 17.0 software (SPSS Inc., Chicago, IL, USA) and MedCalc® (Mariakerke, Belgium) software. The chi-square or Fisher P tests were used to compare qualitative data. Quantitative data were presented as the mean (±standard deviation [SD]) and Student’s t test was used to analyze quantitative data. All data that proved to be statistically significant on univariate analysis were further assessed using multivariate logistic regression. According to the logistic regression findings, the sensitivity, specificity, accuracy, PPV and NPV were calculated using MedCalc® software. A *P* value < 0.05 was considered statistically significant.
